# Dimerization of Neuronal Calcium Sensor Proteins

**DOI:** 10.3389/fnmol.2018.00397

**Published:** 2018-11-02

**Authors:** James B. Ames

**Affiliations:** Department of Chemistry, University of California, Davis, Davis, CA, United States

**Keywords:** calcium, dimer, GCAP1, GCAP2, GCAP5, recoverin, VILIP1, NCS protein

## Abstract

Neuronal calcium sensor (NCS) proteins are EF-hand containing Ca^2+^ binding proteins that regulate sensory signal transduction. Many NCS proteins (recoverin, GCAPs, neurocalcin and visinin-like protein 1 (VILIP1)) form functional dimers under physiological conditions. The dimeric NCS proteins have similar amino acid sequences (50% homology) but each bind to and regulate very different physiological targets. Retinal recoverin binds to rhodopsin kinase and promotes Ca^2+^-dependent desensitization of light-excited rhodopsin during visual phototransduction. The guanylyl cyclase activating proteins (GCAP1–5) each bind and activate retinal guanylyl cyclases (RetGCs) in light-adapted photoreceptors. VILIP1 binds to membrane targets that modulate neuronal secretion. Here, I review atomic-level structures of dimeric forms of recoverin, GCAPs and VILIP1. The distinct dimeric structures in each case suggest that NCS dimerization may play a role in modulating specific target recognition. The dimerization of recoverin and VILIP1 is Ca^2+^-dependent and enhances their membrane-targeting Ca^2+^-myristoyl switch function. The dimerization of GCAP1 and GCAP2 facilitate their binding to dimeric RetGCs and may allosterically control the Ca^2+^-dependent activation of RetGCs.

## Introduction

Intracellular calcium ion (Ca^2+^) is a second messenger in the brain and retina that modulates sensory signal transduction processes (Berridge et al., 2000; Augustine et al., 2003). Sensory stimuli cause changes in cytosolic Ca^2+^ levels that are detected by a family of Ca^2+^-binding proteins called, neuronal calcium sensor (NCS) proteins (Ames et al., 1996; Braunewell and Gundelfinger, 1999; Burgoyne and Weiss, 2001; Burgoyne et al., 2004; Weiss et al., 2010; Ames and Lim, 2012). More than 20 different NCS proteins have been identified so far (Weiss and Burgoyne, 2002; Haynes et al., 2012), including recoverin (Dizhoor et al., 1991) and guanylyl cyclase activating proteins (GCAP1–5; Dizhoor et al., 1994; Palczewski et al., 1994) that regulate visual phototransduction in retinal photoreceptor cells (Palczewski et al., 2000; Ames and Ikura, 2002; Stephen et al., 2008; Ames and Lim, 2012). NCS homologs are also expressed in the brain and spinal cord, such as neurocalcin (Hidaka and Okazaki, 1993), frequenin (NCS-1; Pongs et al., 1993; McFerran et al., 1998), visinin-like proteins (VILIPs; Bernstein et al., 1999; Braunewell and Klein-Szanto, 2009) and hippocalcin (Kobayashi et al., 1992, 1993; Tzingounis et al., 2007).

Recoverin (Dizhoor et al., 1991; Kawamura and Murakami, 1991) is expressed exclusively in retinal rod and cone cells, where it promotes the desensitization of light-excited rhodopsin (Kawamura, 1993; Erickson et al., 1998; Makino et al., 2004) by inhibiting rhodopsin kinase activity in dark-adapted photoreceptors (Calvert et al., 1995; Chen et al., 1995; Klenchin et al., 1995; Komolov et al., 2009). The Ca^2+^-bound form of recoverin forms a dimer in solution (Myers et al., 2013) that binds to rhodopsin kinase (Chen et al., 1995; Klenchin et al., 1995). Recoverin dimerization has been suggested to facilitate the binding of rhodopsin kinase with dimeric rhodopsin (Myers et al., 2013). Recoverin dimerization may also regulate light-dependent activation of phosphodiesterase (Chen et al., 2012) and light-induced disulfide dimerization at Cys39 (Permyakov et al., 2007, 2012; Zernii et al., 2015). Lastly, recoverin appears to have alternative functions in the rod inner segment (Strissel et al., 2005) that are implicated in cancer-associated retinopathy (Polans et al., 1991; Subramanian and Polans, 2004).

GCAP1–5 bind to and activate retinal guanylyl cyclases (RetGCs1 and RetGC2; Dizhoor et al., 1994; Palczewski et al., 1994, 2004). The GCAP1-modulated RetGC1 transduction system also exists in the olfactory bulb (Duda et al., 2001). GCAP1, GCAP2 and GCAP5 each form a dimer in solution (Ermilov et al., 2001; Lim et al., 2017, 2018) that binds to dimeric RetGC1 (Liu et al., 1997; Ramamurthy et al., 2001). The GCAPs activate RetGCs at low Ca^2+^ levels in light activated photoreceptors (Peshenko and Dizhoor, 2006; Lim et al., 2009), whereas Ca^2+^-bound GCAPs inhibit RetGCs at high Ca^2+^ levels in dark-adapted photoreceptors (Dizhoor and Hurley, 1996; Dizhoor et al., 1998). Surprisingly, Ca^2+^-bound GCAP1 can stimulate the odorant surface receptor ONE-GC (Duda et al., 2012a), which raises the question about how GCAP1 dimeric sites can recognize two different target sites existing in RetGC1 and ONE-GC. The Ca^2+^ sensitive activation of RetGCs by GCAPs in the retina promotes the recovery phase of visual excitation, and particular GCAP1 mutants that disrupt the cyclase activation are linked to retinal degenerative diseases (Semple-Rowland et al., 1996; Sokal et al., 1998; Baehr and Palczewski, 2007; Bondarenko et al., 2010; Jiang and Baehr, 2010).

The VILIP1–3; (Braunewell and Klein-Szanto, 2009) are dimeric NCS proteins (Li et al., 2009) that are expressed exclusively in the brain and spinal cord. VILIP1 is localized primarily in the rat hippocampus (Paterlini et al., 2000; Zhao and Braunewell, 2008), where it controls neuronal excitability important for learning and memory (Braunewell et al., 2003; Brackmann et al., 2004). In particular, VILIP1 binds to the α–subunit of the α_4_β_2_ nicotinic acetylcholine receptor (nAChR), which promotes its surface expression and trafficking in oocytes (Lin et al., 2002) and hippocampal neurons (Zhao et al., 2009b). The Ca^2+^-induced surface expression of nAChR promoted by VILIP1 therefore modulates neuronal excitability in hippocampal neurons (Gierke et al., 2008; Zhao et al., 2009a,b) and regulates synaptic plasticity (Braunewell, 2005; Braunewell and Klein-Szanto, 2009).

**Figure 1:**
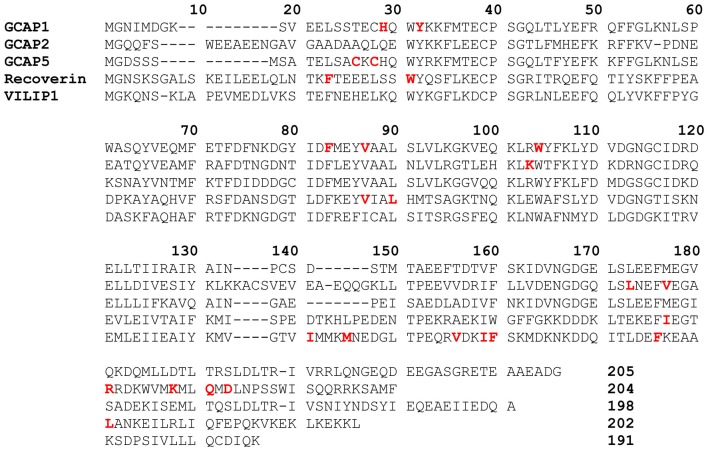
Amino acid sequence alignment of selected neuronal calcium sensor (NCS) proteins. Residues at the domain interfaced are highlighted in bold and red. Swiss Protein Database accession numbers are P46065 (bovine GCAP1), 51177 (bovine GCAP2), Q5MAC8 (zebrafish GCAP5), P21457 (bovine recoverin), P61602 (bovine neurocalcin-δ) and P62760 (human visinin-like protein 1 (VILIP1)).

All NCS proteins contain four EF-hand Ca^2+^-binding motifs (Moncrief et al., 1990; Ikura, 1996), a covalently attached N-terminal myristoyl group, and conserved amino acid residues in the EF-hand motifs, particularly in the Ca^2+^-binding loops (Figure 1). The first EF-hand (EF1) contains a Cys followed by Pro in the binding loop that disables Ca^2+^ binding at this site in all NCS proteins. The second and third EF-hands (EF2 and EF3) both bind Ca^2+^ with high affinity (Cox et al., 1994; Ames et al., 1995). The fourth EF-hand sequence is variable, and Ca^2+^ is able to bind to EF4 in neurocalcin-δ (Ladant, 1995) and GCAPs (Peshenko and Dizhoor, 2007; Stephen et al., 2007) but Ca^2+^ does not bind to EF4 in recoverin (Ames et al., 1995) and VILIPs (Cox et al., 1994; Li et al., 2011). Ca^2+^-binding to EF4 in GCAP1 controls whether GCAP1 can activate or inhibit guanylyl cyclase (Peshenko and Dizhoor, 2007). Residues outside the EF-hand motifs are generally not conserved and may play a role in target recognition (Zernii et al., 2011).

N-terminal myristoylation anchors some NCS proteins to cellular membranes by a mechanism termed, Ca^2+^-myristoyl switch (Dizhoor et al., 1992; Kobayashi et al., 1993; Ladant, 1995). Myristoylated recoverin binds to retinal disc membranes at high Ca^2+^ levels in dark-adapted photoreceptors (Zozulya and Stryer, 1992; Dizhoor et al., 1993; Dell’Orco et al., 2012), whereas unmyristoylated recoverin is localized in the cytosol (Zozulya and Stryer, 1992; Dizhoor et al., 1993). Likewise, myristoylated forms of neurocalcin (Ladant, 1995), hippocalcin (Kobayashi et al., 1993) and VILIPs (Li et al., 2011) each exhibit Ca^2+^-induced localization at the plasma membrane in neurons. The attached fatty acyl group interacts quite differently with each NCS protein as seen in the structures for Ca^2+^-free recoverin (Tanaka et al., 1995), NCS1 (Lim et al., 2011), GCAP1 (Lim et al., 2016) and VILIP3 (Li et al., 2016). Thus, N-terminal myristoylation serves to fine tune the tertiary structure of each NCS protein in a unique way to promote functional diversity (Ames and Lim, 2012). Recoverin’s Ca^2+^-myristoyl switch may control its light-induced movement into the rod inner segment (Strissel et al., 2005). GCAP proteins are also myristoylated (Palczewski et al., 1994; Frins et al., 1996; Olshevskaya et al., 1997), but do not possess a functional Ca^2+^-myristoyl switch (Olshevskaya et al., 1997; Hwang and Koch, 2002). Instead the N-terminal myristoyl group remains sequestered inside GCAP1 in both Ca^2+^-free and Ca^2+^-bound states (Hughes et al., 1998; Lim et al., 2009) as demonstrated in the crystal structure of Ca^2+^-bound GCAP1 (Stephen et al., 2007) and NMR structure of the Ca^2+^-free activator state (Lim et al., 2016).

**Figure 2:**
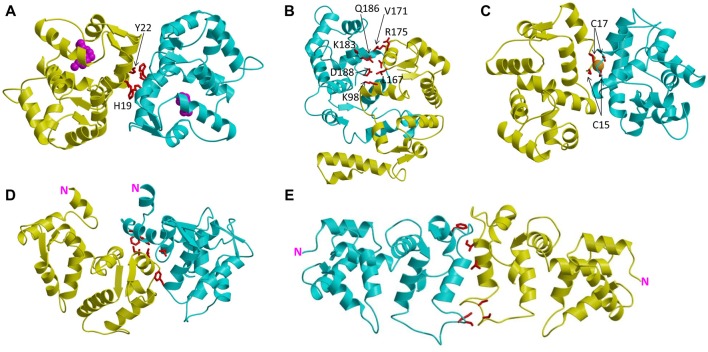
Main chain structures of dimeric NCS proteins: Ca^2+^-free/Mg^2+^-bound GCAP1 **(A)**, Ca^2+^-free GCAP2 **(B)**, Fe^2+^-bound GCAP5 **(C)**, Ca^2+^-bound recoverin **(D)** and Ca^2+^-bound VILIP1 **(E)**. The two separate dimer subunits are colored yellow and cyan. Amino acids at the dimer interface are highlighted red. Fe^2+^ bound to GCAP5 is orange, and N-terminal myristoyl group in GCAP1 is magenta.

In this review article, I discuss the recent atomic-resolution structures of dimeric forms of recoverin (Myers et al., 2013), GCAP1 (Lim et al., 2018), GCAP2 (Pettelkau et al., 2012), GCAP5 (Lim et al., 2017) and VILIP1 (Li et al., 2011) that each adopt very different quaternary structures (Figure 2). While the tertiary structures of each monomeric subunit are somewhat similar, the distinct quaternary structures and unique subunit packing arrangement at each dimer interface may play a role in facilitating target recognition and specificity.

## NCS Proteins Have Distinct Dimeric Structures

### GCAP1 Forms a Symmetric and Functional Dimer

**Figure 3:**
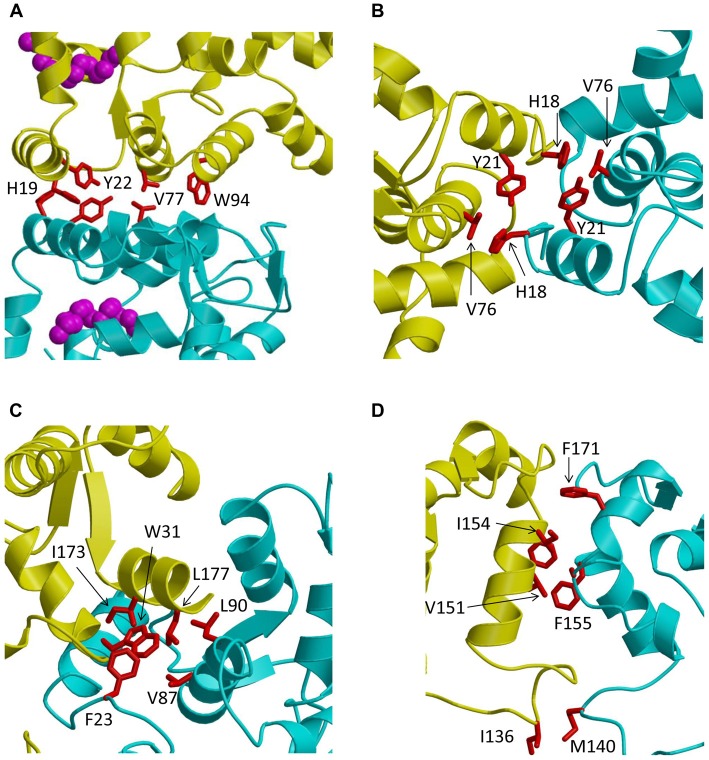
Close-up views of the dimerization interface in GCAP1 **(A)**, GCAP5 **(B)**, recoverin **(C)** and VILIP1 **(D)**. The two dimer subunits are colored yellow and cyan. Hydrophobic amino acids at the dimer interface are highlighted red.

Recent NMR (Lim et al., 2009, 2013, 2016) and EPR double electron-electron resonance (DEER; Lim et al., 2018) studies reveal that GCAP1 exists as a dimer in solution. The GCAP1 dimerization is Ca^2+^-independent and Ca^2+^-binding to GCAP1 does not appear to cause large changes in the dimer quaternary structure (Lim et al., 2018). A structural model of the GCAP1 dimer was determined recently by DEER (Lim et al., 2018; Figure 2A). The GCAP1 dimer is symmetric (Figure 2A), and is stabilized by hydrophobic contacts at the dimer interface (Figure 3A). The most noteworthy intermolecular contacts involve hydrophobic residues, H19, Y22, V77 and W94 (Figure 3A). In particular, the methyl side-chain atoms of V77 each contact one another at the dimer interface and therefore explain why the V77E mutation dramatically weakens GCAP1 dimerization (Lim et al., 2016). The GCAP1 dimer is further stabilized by aromatic side chains of F73 and W94 that form intermolecular contacts at the dimer interface (Lim et al., 2018). Individual point mutations at the dimer interface in GCAP1 (H19A, Y22A, F73A, V77E and W94A) each weaken the dimerization dissociation constant by more than 10-fold and completely abolish the activation of RetGC by GCAP1 (Lim et al., 2018). Thus, the hydrophobic contacts at the GCAP1 dimer interface (Figure 3A) are essential for both its dimerization and activation of RetGCs. This implies that GCAP1 dimerization may be important for activating RetGC1, which itself is a dimer (Liu et al., 1997; Ramamurthy et al., 2001). Therefore, it is tempting to speculate that the GCAP1 dimer (Figure 2A) may bind to dimeric RetGC1 to form a 2:2 complex. This binding of the GCAP1 dimer is proposed to induce an allosteric conformational change in the RetGC1 dimer in order to modulate the cyclase activity. The allosteric regulation of RetGC activity may involve quaternary structural changes in the 2:2 complex akin to quaternary structural changes that regulate O_2_ binding to hemoglobin (Monod et al., 1965).

### GCAP2 Forms an Asymmetric Dimer With a Polar Dimer Interface

GCAP2 forms a stable dimer in solution (Ames et al., 1999; Ermilov et al., 2001), and the original NMR structure of GCAP2 (Ames et al., 1999) was determined in the presence of detergent that dissociated the dimer into a stably folded monomer. There is some dispute about the Ca^2+^-dependence of the GCAP2 dimerization. The original work by Ermilov et al. (2001) determined that GCAP2 forms a dimer only in the Ca^2+^-free state, and the Ca^2+^-bound GCAP2 was shown to be monomeric. However, a more recent study suggested that GCAP2 can form a dimer in both the Ca^2+^-free and Ca^2+^-bound states (Pettelkau et al., 2013). A structural model of the GCAP2 dimer (in both Ca^2+^-free and Ca^2+^-bound states) was determined recently by mass spectrometry (Pettelkau et al., 2013). Ca^2+^-binding to GCAP2 does not affect the dimer quaternary structure (Pettelkau et al., 2013), similar to the Ca^2+^-independent dimer quaternary structure of GCAP1 (Lim et al., 2018). However unlike GCAP1, the GCAP2 dimer is asymmetric (Figure 2B). The residues at the GCAP2 dimer interface (residues K98, L167, V171, R175, K183, Q186, D188 highlighted red in Figure 2B) are not conserved and are completely unrelated to the residues at the GCAP1 interface (highlighted red in Figure 3A). Surprisingly, the GCAP2 interface involves mainly polar and charged residues in contrast to the exclusively hydrophobic interface in GCAP1. The positively charged side-chain atoms of R175 in GCAP2 (yellow colored chain in Figure 2B) are within 5 Å of the polar side-chain atoms of Q186 in the opposite chain (cyan in Figure 2B), and the positively charged side-chain atoms of K98 (yellow chain in Figure 2B) are less than 4 Å from the side chain carboxylate atoms of D188 in the opposite chain (cyan in Figure 2B). These intermolecular contacts at the GCAP2 dimer interface are not conserved in GCAP1 and may explain why the GCAP2 dimer (Figure 2B) is structurally quite different from the GCAP1 dimer (Figure 2A). The dramatically different quaternary structures and dimerization interface for GCAP2 compared to GCAP1 might also explain their functional differences (Duda et al., 2012b; Peshenko et al., 2015).

### GCAP5 Dimerization Is Bridged by Fe^2+^

Zebrafish photoreceptors contain specific GCAP homologs (GCAP3–5; Imanishi et al., 2004; Rätscho et al., 2009) that are not expressed in mammals. The amino acid sequence of the zebrafish homolog called GCAP5 is the most divergent compared to the amino acid sequences of mammalian GCAP1 and GCAP2 (Figure 1). Two non-conserved Cys residues in GCAP5 (Cys 15 and Cys17) were shown recently to ligate Fe^2+^ (Lim et al., 2017). Fe^2+^-binding to GCAP5 serves as a potent inhibitor and the Fe^2+^-bound GCAP5 is unable to activate RetGC at low Ca^2+^ levels in light-adapted photoreceptors, suggesting that Fe^2+^ binding to GCAP5 may serve as a redox sensor for phototransduction in zebrafish photoreceptors (Lim et al., 2017). Structurally, the Fe^2+^ binding by Cys15 and Cys17 bridges two GCAP5 molecules into a [Fe(SCys)_4_] dimeric complex (Lim et al., 2017) like that observed previously in two-iron superoxide reductases (deMaré et al., 1996; Min et al., 2001; Emerson et al., 2003). The GCAP5 dimer has a symmetric structure (Figure 2C) somewhat similar to that of GCAP1 (Figure 2A). The GCAP5 dimer interface contains hydrophobic residues (H18, Y21 and V76 in Figure 3B) that are conserved in the GCAP1 dimer (Figure 3A). However unlike GCAP1, the GCAP5 dimer contains a single Fe^2+^ bound at the dimer interface that is ligated by Cys15 and Cys17 in both dimer subunits (colored yellow and cyan in Figure 2C) of the symmetric GCAP5 dimer. The four cysteinyl thiolate groups that ligate the bound Fe^2+^ (Figure 2C) are similar in structure to the four Cys residues found in the Cys4 zinc finger motif that binds to Zn^2+^ (Tang et al., 2014). The structural similarity to the Cys4 zinc finger suggests that GCAP5 may also bind to Zn^2+^ in place of Fe^2+^. Zn^2+^ is transported into retinal photoreceptor cells and has been suggested to play a role in phototransduction (Redenti et al., 2007). It is tempting to speculate that GCAP5 might serve as a Zn^2+^ sensor in the zebrafish photoreceptor. Future studies are needed to probe whether Zn^2+^ can bind to GCAP5 and test whether Zn^2+^ binding to GCAP5 (like Fe^2+^ binding) can also regulate zebrafish RetGCs during visual phototransduction.

### Ca^2+^-Induced Dimerization of Ca^2+^-Myristoyl Switch Proteins

**Figure 4:**
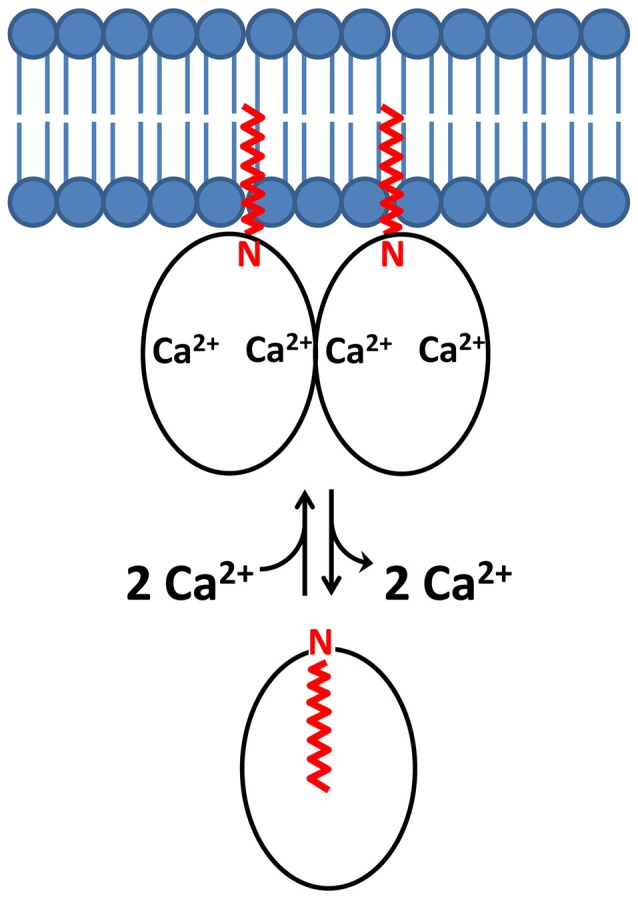
Schematic model of a dimeric Ca^2+^-myristoyl switch with a dual myristate membrane anchor. N-terminal myristoyl group is highlighted in red and lipid bilayer in blue.

The Ca^2+^-myristoyl switch proteins, recoverin (Myers et al., 2013) and VILIP1 (Li et al., 2011) both exhibit Ca^2+^-induced dimerization that enhances their membrane anchoring. The dimeric structure of Ca^2+^-bound recoverin (Figure 2D) places both of its exposed N-terminal myristoyl groups (highlighted magenta in Figure 2D) pointing in the same direction to serve as a dual pronged myristate membrane anchor (Figure 4). The recoverin dimer is stabilized mostly by hydrophobic intermolecular contacts (Figure 3C). In essence, the dimer interface is formed by the exposed hydrophobic residues in the exiting helix of EF4 (residues I173 and L177) that fit snuggly into the exposed hydrophobic groove between EF1 and EF2. Aliphatic side chain atoms of I173 and L177 from EF4 (yellow chain in Figure 3C) make intermolecular contacts with aromatic side chain atoms of F23 and W31 (cyan chain in Figure 3C). Additional intermolecular contacts are formed by side chain atoms of L90 and L177. The N-terminal domain residues (F23, W31, V87 and L90) at the dimer interface are the same residues that interact with the sequestered myristoyl group in Ca^2+^-free recoverin (Tanaka et al., 1995; Ames et al., 1997; Ames and Lim, 2012). The Ca^2+^-induced extrusion of the myristoyl group causes the exposure of these residues (F23, W31, V87 and L90), making them accessible to promote dimerization of the Ca^2+^-bound protein. The Ca^2+^-induced dimerization of recoverin enhances membrane binding by creating a dual pronged myristoyl anchor (Figure 4). The membrane anchored recoverin dimer bound to two rhodopsin kinase molecules in the dark may serve to place the two kinase molecules in close proximity of dimeric rhodopsin and therefore facilitate their rapid binding upon light activation (Ames et al., 2006; Myers et al., 2013).

The dimeric structure of VILIP-1 contains two protein subunits attached at their exposed C-terminal ends, forming an elongated structure (see yellow and cyan chains in Figure 2D). The exposed helices of EF4 are packed against each other at the dimer interface, forming an intermolecular four helix bundle. The VILIP1 dimer interface is almost entirely hydrophobic (Figure 3D). Exposed residues on the entering helix of EF4 (V151 and I154) in the yellow chain of Figure 2D make intermolecular contacts with exposed hydrophobic residues (F155 and F171) in the cyan colored chain. Additional intermolecular hydrophobic contacts are formed by I136 and M140 located in the region between EF3 and EF4. The intermolecular hydrophobic contacts are essential for VILIP1 dimerization as demonstrated by mutants (I136G, V151G and F155G) that each weaken the dimerization affinity (Li et al., 2011). The VILIP1 dimer structure has its N-terminal myristoyl group from each dimer subunit pointing upward in the same direction to serve as a dual-pronged anchor for targeting VILIP-1 to membranes (Figure 4). The opposite end of the VILIP1 dimer contains an exposed hydrophobic crevice in the N-terminal domain (residues F22, W30, L43, F48, I51, Y52, F55, F56, F72, F82, F85, I86, A88, L89) that are suggested to interact with hydrophobic segments of target proteins (Li et al., 2011).

## Functional Implications of NCS Dimerization

### Recoverin and VILIP1 Dimers Enhance Membrane Targeting Affinity

The Ca^2+^-bound dimeric structures of recoverin (Figure 2C) and VILIP1 (Figure 2D) have their N-terminal myristoyl groups pointing in the same direction toward the membrane surface (Figure 4). The juxta positioning of the two myristoyl groups creates a dual pronged membrane anchor that entropically enhances membrane binding. Since the effect of dimerization is multiplicative, a dimeric myristoyl switch protein is predicted to bind to lipid bilayer membranes with 10^4^-fold higher affinity (*K*_d_ = 10^–8^ M) compared to the affinity of a monomeric Ca^2+^-myristoyl switch (*K*_d_ = 10^–4^ M; Peitzsch and McLaughlin, 1993; Kim et al., 1994; Dell’Orco et al., 2012). In other words, the membrane binding dissociation constant of the dimer (*K*_d_(dimer)) is equal to the square of the dissociation constant of the monomer: *K*_d_(dimer) = *K*_d_(monomer)^2^ = (10^–4^ M)^2^ = 10^–8^ M. Thus, the membrane binding affinity of Ca^2+^-myristoyl switch proteins is predicted here to be dramatically enhanced by the combined effect of both Ca^2+^-binding and protein dimerization. Dimerization of Ca^2+^-myristoyl switch proteins may also entropically enhance its binding to dimeric protein targets, as was suggested for the binding of dimeric Ca^2+^-bound recoverin to dimeric rhodopsin (Myers et al., 2013).

### GCAP1 Dimerization in Retinal Photoreceptors

An important unresolved question in visual phototransduction is how the Ca^2+^-free GCAP proteins are able to specifically bind and activate RetGC in light-adapted photoreceptors, and conversely how Ca^2+^-bound GCAPs inhibit RetGC in dark-adapted photoreceptors. The crystal structure of the Ca^2+^-bound GCAP1 inhibitory state (Stephen et al., 2007) is overall similar to the recent NMR structure of the Ca^2+^-free GCAP1 activator state (Lim et al., 2016). Although, the Ca^2+^-induced changes in tertiary structure for GCAP1 appear moderately small, these small tertiary structural changes may promote a functional change in the quaternary structure of the GCAP1 dimer that in turn could modulate the quaternary structure of the RetGC1 dimer in order to allosterically regulate cyclase activity. In other words, small changes in tertiary structure may result in much larger changes in quaternary structure in order to amplify the response. Consistent with this prediction, mutations in GCAP1 (H19E, Y22E, F73E, V77E and W94E) that each weaken dimerization also abolish activation of the cyclase (Lim et al., 2018). These mutants indicate that GCAP1 dimerization is necessary and sufficient to activate RetGC, and furthermore suggests that a pre-formed GCAP1 dimer may facilitate its binding to the dimeric RetGC and thus stabilize a high affinity 2:2 target complex.

An alternative view is that the GCAP1 dimer that has been detected in solution and in the absence of RetGC may not necessarily exist in the presence of RetGC, because residues in the GCAP1 dimer interface (Figure 3A) appear to overlap with residues that interact with RetGC (Peshenko et al., 2014). In this scenario, the residues at the GCAP1 dimerization site may prefer to interact with RetGC (rather than itself) in the presence of saturating RetGC, and the binding of RetGC to GCAP1 in this case would be expected to prevent GCAP1 dimerization. To distinguish whether GCAP1 dimerization facilitates or opposes RetGC binding, future studies are needed to probe whether or not the structure of the GCAP1 dimer (Figure 2A) will remain intact when GCAP1 is bound to RetGC.

## Conclusion

The dimerization of NCS proteins could help explain how these highly conserved proteins adopt distinctive structures that recognize many different targets. Recent structures of dimeric forms of GCAP1, GCAP2, GCAP5, recoverin and VILIP1 each reveal a unique quaternary structure at the dimer interface. GCAP1 forms a symmetric dimer that consolidates key residues for interacting with RetGCs, whereas GCAP2 forms an asymmetric dimer. The dimerization of GCAPs may facilitate allosteric regulation of its dimeric target protein (RetGC), which may help explain the steep Ca^2+^-dependent regulation of RetGC. Dimerization of both recoverin and VILIP1 creates a dual pronged myristate membrane anchor that enhances membrane targeting and may facilitate recognition of dimeric membrane-bound targets.

## Author Contributions

JA wrote and conceived the entire manuscript.

## Conflict of Interest Statement

The author declares that the research was conducted in the absence of any commercial or financial relationships that could be construed as a potential conflict of interest.
